# Effects of Adipose-Derived Stem Cell Extract on Peripheral Nerve Cells

**DOI:** 10.7759/cureus.94180

**Published:** 2025-10-09

**Authors:** Yuzo Imai, Naotaka Kishimoto, Yuhei Koyama, Kazunori Sango, Kosei Takeuchi, Manabu Yamazaki, Jun-ichi Tanuma, Simon D Tran, Kenji Seo

**Affiliations:** 1 Division of Dental Anesthesiology, Faculty of Dentistry, Graduate School of Medical and Dental Sciences, Niigata University, Niigata, JPN; 2 Diabetic Neuropathy Project, Department of Diseases and Infection, Tokyo Metropolitan Institute of Medical Science, Tokyo, JPN; 3 Department of Medical Cell Biology, Aichi Medical University, Nagoya, JPN; 4 Division of Oral Pathology, Faculty of Dentistry, Graduate School of Medical and Dental Sciences, Niigata University, Niigata, JPN; 5 Faculty of Dental Medicine and Oral Health Sciences, McGill University, Montreal, CAN

**Keywords:** adipose-derived stem cells, cell extract, nerve injury, nerve regeneration, peripheral nerve

## Abstract

Adipose-derived stem cells (ADSCs) can be obtained from adipose tissue, which is considered clinically dispensable. ADSCs have the ability to differentiate not only into adipocytes and osteoblasts but also into various other cell types, such as nerve cells and cardiomyocytes. However, the clinical application of ADSCs in stem cell therapy is hampered by the risk of transplant rejection and the need for facilities for their storage and transportation. In comparison, cell extracts (CEs) obtained from stem cells by freeze-thawing and lysis are less tumorigenic and immunogenic. However, there are currently no studies on the application of ADSC-derived CEs (ADSC-CEs) in peripheral nerve regeneration. Therefore, in this study, we investigated the effects of ADSC-CEs on proliferation and neurite extension in peripheral nerve cells. ADSCs were harvested from the inguinal region of mice, and ADSC-CEs were obtained following repeated freeze-thawing of ADSCs. We examined the effects of the ADSC-CEs, added to the culture medium, on glial fibrillary acidic protein (GFAP) expression and proliferation in Schwann cells. Moreover, we examined the effects of the ADSC-CEs on neurite length in DRG neurons and PC12D cells. ADSC-CEs stimulated the proliferation of Schwann cells, elevated GFAP expression in these cells, and promoted the elongation of DRG neuron and PC12D cell projections. Notably, heat treatment of the ADSC-CEs abolished these effects. Together, these findings suggest that ADSC-CEs may have therapeutic application in peripheral nerve regeneration.

## Introduction

Peripheral nerve injury can drastically reduce the quality of life [1,2]. In cases of severe nerve damage, surgical treatment with autologous nerve grafts or nerve conduits is used because adequate tissue regeneration cannot be obtained otherwise [3]. However, autologous nerve grafting is indicated only in a limited number of cases because of the need to harvest a segment of the patient’s healthy nerve and differences in the size of the donor and recipient nerves at the sites of transplant [3]. In addition, when nerve conduit repair is used to repair a nerve with a large defect length or diameter, a neuroma may form [4]. As an alternative treatment, regenerative medicine using stem cells, such as adipose-derived stem cells (ADSCs), is currently under intensive investigation [3].

Peripheral nerves are anatomically composed of neurons, myelin sheaths, and connective tissues [5]. Schwann cells are glial cells that are arranged along axons and form myelin sheaths [3]. Diagnosis-related group (DRG) neurons are sensory neurons located in the DRGs of the spinal cord and are part of both the peripheral and central nervous systems [6]. When peripheral nerves are injured, Schwann cells phagocytose damaged myelin and secrete neurotrophic factors and cytokines. Secreted cytokines mobilize macrophages and promote the phagocytosis of myelin by these cells [3]. Schwann cells multiply and form bands of Büngner that participate in nerve regeneration from the site of injury to the target region [7]. Neurotrophic factors secreted by Schwann cells increase the size of neuronal cell bodies and promote dendrite outgrowth [3].

ADSCs can be obtained from adipose tissue, which is often considered clinically dispensable. Moreover, ADSCs have the advantage of high proliferative potential and low invasiveness at the time of collection [8], and they have the ability to differentiate into numerous cell types, including adipocytes, osteoblasts, chondrocytes, neural cells, cardiomyocytes, and hepatocytes [9]. In addition, the peripheral nerve regeneration-promoting effect of ADSCs is not affected by the age of the donor [10]. However, the clinical application of stem cell therapy is hampered by the risk of transplant rejection and the need for facilities for the storage and transportation of stem cells [11]. Nonetheless, in mouse models of nerve injury, ADSCs have been reported to promote peripheral nerve regeneration by releasing angiogenesis-related factors, such as vascular endothelial growth factor (VEGF) and hepatocyte growth factor (HGF), in vivo [10,12]. Therefore, secreted factors produced by stem cells might be key to their ability to promote nerve regeneration and repair [11]. Accordingly, regenerative medicine using stem cell-derived extracts (CEs) without the use of stem cells is currently a focus of study [11].

CEs obtained from stem cells by freeze-thawing and lysis are theoretically less tumorigenic and immunogenic because of the lack of cellular structures [11,13]. A CE derived from bone marrow cells has been reported to restore irradiated salivary gland function even after cryopreservation for more than one year or freeze-drying for two months [14]. Accordingly, there are fewer storage and transportation limitations compared with stem cell-based therapy. It has been reported that CEs derived from splenocytes or ADSCs can restore salivary gland function after irradiation when administered through the tail vein in mice [15]. CEs derived from lip gland stem cells [11] or bone marrow cells [16] have also been reported to restore salivary gland function when administered through the tail vein in mice. Moreover, a CE derived from bone marrow cells has been reported to restore cardiac function after myocardial infarction [17]. These reports suggest that CEs might be useful for regeneration therapy for various tissues and organs. Fang and colleagues [18] reported that a CE from bone marrow cells contains angiogenesis-related factors, including nerve growth factor (NGF) and VEGF, that improve salivary gland function after irradiation. However, there are currently no studies on the application of ADSC-derived CEs (ADSC-CEs) in peripheral nerve regeneration.

We hypothesized that proteins produced by ADSC-CEs might activate peripheral nerve cells. Therefore, in this study, we investigated the effects of ADSC-CEs on the proliferation and neurite extension of peripheral nerve cells.

This article was previously presented as a meeting abstract at the 2021 Neuroscience 50th annual meeting of the Society for Neuroscience on November 8, 2021.

## Materials and methods

Isolation of adipose-derived stem cells

**Figure 1:**
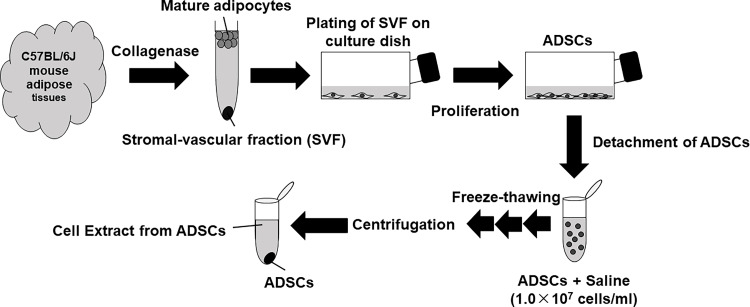
Establishment of adipose-derived stem cells (ADSCs) and preparation of cell extract (CE) The image is created by the author. Adipose tissue from the inguinal region of mice was cut into small pieces and treated with collagenase solution. The pieces were then centrifuged to separate the stromal vascular fraction (SVF), and ADSCs were established by in vitro culture. The ADSCs were suspended in saline (1.0 × 10^7^ cells/ml), and the suspension was frozen and thawed three times. The supernatant was collected by centrifugation and used as CE derived from ADSCs (ADSC-CEs) for the experiment. This figure was prepared by modifying the figure published by Kishimoto et al. (*Oral Dis*, 2018; 24; 1161–1167).

Animal experiments in this study were approved by the Animal Experiment Ethics Review Committee of Niigata University (approval number: SA00930). First, mice were euthanized using sevoflurane (Pfizer, New York, NY, USA) and a CO₂ device, and adipose tissue was harvested from the inguinal region (Figure 1). Appendix shows the experimental protocol. The adipose tissues were washed thrice with Dulbecco’s phosphate-buffered saline (PBS; Nacalai Tesque, Kyoto, Japan) supplemented with 1% penicillin/streptomycin (FUJIFILM Wako Chemicals, Osaka, Japan) and then dissociated using 0.1% collagenase type 1 solution (037-17603; FUJIFILM Wako Chemicals) for 1 hour at 37°C under a 5% CO₂/95% air atmosphere. After 1 hour, the tissue was filtered through a 100 µm filter (BM Equipment Co., Ltd., Tokyo, Japan) and centrifuged (1,000 rpm, 4°C, 5 min) to separate the stromal vascular fraction (SVF), which was then seeded into 75 cm² flasks (Corning Inc., Corning, NY, USA). After assessing cell viability the next day, non-adherent cells were removed by changing the culture medium. The basic medium was Dulbecco’s modified Eagle medium (DMEM; Nacalai Tesque, Code No. 14249-95) containing 20% fetal bovine serum (FBS; Thermo Fisher Scientific, Waltham, MA, USA) and 1% penicillin/streptomycin. The medium was changed twice a week, and the proliferating cells were harvested as ADSCs. When the ADSCs were approximately 80% confluent, they were passaged with 0.125% trypsin solution (0.25% w/v trypsin solution with phenol red; FUJIFILM Wako Chemicals). Third-passage ADSCs were used for subsequent experiments.

Adipocyte differentiation

The multipotency of adipose-derived stem cells (ADSCs) was evaluated using the Mouse Mesenchymal Stem Cell Functional Identification Kit (R&D Systems, Minneapolis, MN, USA), following the manufacturer's instructions. The ADSCs were seeded into a 24-well plate at a density of 3.7 × 10^4^ cells per well and cultured overnight at 37°C in an atmosphere containing 5% CO₂. The culture medium consisted of αMEM basal medium supplemented with 10% FBS and 1% penicillin, streptomycin, and glutamine. The next day, once the cells had reached 100% confluence, the medium was replaced with an adipogenic induction medium (αMEM basal medium supplemented with 1% adipogenic supplement). The adipogenic medium was replaced every three days, and after 14 days, the adipocytes were fixed, and double fluorescent immunostaining was performed using FABP4 and Hoechst 33342.

Osteoblast differentiation

ADSCs were seeded into a 24-well plate (7.4 × 10³ cells/well) and cultured overnight at 37°C in a 5% CO₂ environment in αMEM basal medium. The next day, when the cells reached 70% confluence, the medium was replaced with bone induction medium (αMEM basal medium supplemented with 5% osteogenic supplement). This medium was replaced every three days, and after 21 days, the osteoblasts were fixed, and double immunofluorescence staining was performed using osteopontin and Hoechst 33342.

Chondrocyte differentiation

ADSCs were treated with trypsin, and a suspension of 2.5 × 10^5^ cells was transferred to a 15 ml centrifuge tube. The tube was then centrifuged at 200 × g at room temperature for 5 min. The medium was removed, and the cells were resuspended in 1.0 ml DMEM/F12 basal medium (99% DMEM/F12 supplemented with 0.5% ITS supplement and 0.5% penicillin-streptomycin-glutamine) and then centrifuged again (200 × g at room temperature for 5 min). The medium was removed once more, and the cells were resuspended in chondrogenic medium (DMEM/F12 basal medium supplemented with 1% chondrogenic supplement), after which they were cultured in an environment of 37°C and 5% CO₂. The medium was changed every three days. After 21 days, the cells were fixed and subjected to double immunofluorescence staining using collagen II and Hoechst 33342.

Preparation of the ADSC-CE

The ADSC-CE was prepared according to Fang et al. [15] (Figure 1). ADSCs were suspended in saline (Otsuka, Tokyo, Japan) at a concentration of 1 × 10^7^ cells/ml and frozen at −80°C. The cell suspension was then freeze-thawed three times and centrifuged (17,000 rpm, 4°C, 30 min). The suspension was then passed through a 100 µm filter (BM Equipment Co., Ltd.). The resulting supernatant, termed the ADSC-CE, was collected and frozen at −80°C. The total protein concentration in the ADSC-CEs was determined with the Pierce BCA Protein Assay Kit (23225; Thermo Fisher Scientific). ADSC-CEs were diluted five-fold with saline, and the albumin standard (BSA) was diluted with saline, as described in the package insert (final concentration range: 0-2,000 µg/ml). These solutions were added to 96-well plates (Nippon Genetics Co., Ltd., Tokyo, Japan) at 25 µl each. Working reagent (A:B = 1:50) was then added at 200 µl per well. Next, the samples were incubated in the dark (37°C, 30 min). After 10 min at room temperature, the absorbance (570 nm) was measured using an absorbance microplate reader (Thermo Fisher Scientific) to determine total protein concentrations.

Heat treatment of ADSC-CEs

ADSC-CEs were heated as described in Fang et al. [18]. The lids of ring-lock tubes (BM Equipment Co., Ltd.) were covered with parafilm (Bemis Co., Ktd., Neenah, WI, USA) to prevent evaporation and then heated for 60 min at 95°C using a heater (Corning). The tubes were then centrifuged (13,500 rpm, 4°C, 15 min), and the supernatant, termed the heated ADSC-CE, was collected. The total protein concentration was determined with the Pierce BCA Protein Assay Kit (Thermo Fisher Scientific).

Investigation of the effects of ADSC-CEs

Schwann Cell Proliferation

Immortalized rat Schwann cells (IFRS1; Code No. SWN-IFRS1C, Cosmo Bio, Tokyo, Japan) were cultured according to the package insert (Cosmo Bio IFRS1 datasheet) [19]. The cells were thawed and seeded into 75 cm² flasks (Corning Inc.) and then cultured in basic medium (SWNMR; Cosmo Bio). The medium was changed twice a week. When approximately 80% confluent, the cells were passaged with 0.125% trypsin solution (FUJIFILM Wako Chemicals) and used in subsequent experiments.

IFRS1 cells were seeded into 96-well plates (Nippon Genetics Co., Ltd) at 5.0 × 10^3^ cells/well in 100 µl of medium and incubated for 24 h at 37°C under a 5% CO₂/95% air atmosphere. After 24 hours, the medium was replaced with 100 µl of basic medium (control medium) or basic medium supplemented with ADSC-CEs (ADSC-CEs added to the medium to a protein concentration of 6.5, 12.5, 25, or 50 µg/ml). The number of Schwann cells was counted on days zero, two, and five of incubation. Briefly, after removing the culture medium, 100 µl of DMEM containing 10% MTT (345-01821; FUJIFILM Wako Chemicals) was added and incubated for 3 hours under light-shielded conditions. The MTT solution was then removed, and 100 µl of dimethyl sulfoxide (043-02716; FUJIFILM Wako Chemicals) was added to each well. After incubation on a shaker (TAITEK, Saitama, Japan) for 10 min at room temperature under light-shielded conditions, the absorbance (570 nm) was measured using an absorbance microplate reader (Thermo Fisher Scientific). The same procedure was used to evaluate the effect of heated ADSC-CEs on Schwann cell proliferation.

GFAP Expression in Schwann Cells

IFRS1 cells were seeded into 12-well plates (Nippon Genetics Co., Ltd.) at a density of 1.0 × 10^5^ cells/ml per well and incubated at 37°C under a 5% CO2/95% air atmosphere until approximately 80% confluent. Then, the culture medium was removed, and 1 ml of basic medium (control medium) or basic medium supplemented with ADSC-CEs (ADSC-CEs added to the medium to a protein concentration of 12.5 µg/ml) was added to each well. After three, five, or seven days, the culture medium was removed, and the cells were washed twice with PBS (Nacalai Tesque). Then, the cells were incubated in RIPA buffer (08714-04; Nacalai Tesque) at a density of 0.5-5.0 × 10^7^ cells/ml for 5 min on a shaker. The cell suspension was then collected, incubated on ice for 15 min, and centrifuged (10,000 × g, 4°C, 10 min). The supernatant was then collected and stored at −80°C until assay. The GFAP concentration in the collected supernatant was then measured using the Human GFAP ELISA Kit (ab223867; Abcam, Cambridge, UK) according to the package insert. The absorbance (450 nm) was measured using an absorbance microplate reader (Thermo Fisher Scientific). The effect of heated ADSC-CEs on GFAP expression in Schwann cells was evaluated using the same procedure.

Neurite-Bearing DRG Neurons

According to the protocol in Sango et al. [20], DRG neuron cultures were established from 10-12-week-old SD rats (The Jackson Laboratory Japan, Kanagawa, Japan). Briefly, the rats were euthanized using sevoflurane (Pfizer, New York, USA) and a CO₂ apparatus, and the spinal cord was removed. The dorsal root ganglia were isolated under a stereomicroscope (STEMI 305; ZEISS, Oberkochen, Germany) and treated with 2 mg/ml collagenase solution (Collagenase Type III; Worthington, Lakewood, NJ, USA) for 2 h at 37°C and then washed twice with 1× Hank’s balanced salt solution (HBSS; Sigma-Aldrich, St. Louis, MO, USA). The cells were then treated with 2.5 mg/ml trypsin (type I, bovine pancreas; Sigma-Aldrich) for 15 minutes at 37°C, and then 50 µg/ml trypsin inhibitor (Sigma-Aldrich) was added. The dorsal root ganglia were completely dissociated by pipetting, and the lysate was layered in 30% Percoll solution (Sigma-Aldrich) diluted in HBSS and centrifuged (1,000 rpm, 4°C, 5 min). The supernatant was discarded, and the cell pellet was resuspended in DMEM/F12 medium (1130-032; Thermo Fisher Scientific) containing 10% FBS and 1% penicillin/streptomycin. The cells were seeded at a density of 3 × 103 cells/well in a 200 µl volume in eight-well chamber slides (177445, Thermo Fisher Scientific) treated with 10 µg/ml poly-L-lysine (P1524-25MG; Sigma-Aldrich).

Based on the method of Sango et al. [20], 24 hours after isolation of DRG neurons (day zero), the serum-containing medium (DMEM/F12 containing 10% FBS and 1% penicillin/streptomycin) was replaced with basic (control) medium (DMEM/F12 containing 2% B27 (17504-044; Thermo Fisher Scientific) and 1% penicillin/streptomycin) or basic medium supplemented with ADSC-CEs to a protein concentration of 6.25, 12.5, 25, or 50 µg/ml. After 1 hour, DRG neurons in each well were counted under a phase-contrast microscope (OLYMPUS, Tokyo, Japan). DRG neurons with large and bright cell bodies (approximately 20-30 µm in diameter) were considered viable cells and distinguished from other cells (Schwann cells and fibroblasts) according to the report of Sango et al. [21]. Neurite-bearing cells [20], i.e., cells with processes longer than their cell bodies, were counted at one, three, five, and seven days after medium exchange and expressed as a percentage of the total number of DRG neurons (100%). The same procedure was used to evaluate the effect of heated ADSC-CEs on the percentage of neurite-bearing cells.

Length of Neurites of DRG Neurons

According to the method of Sango et al. [21], 24 hours after isolation of DRG neurons (day zero), the serum-containing medium was replaced with 250 µl of basic (control) medium or basic medium containing ADSC-CEs added to a protein concentration of 12.5 µg/ml. Then, four days later, DRG neurons were fixed with 4% paraformaldehyde in PBS for 15 min, followed by permeabilization with 0.2% Triton X-100 in PBS for 15 min. The cells were blocked with 2% normal goat serum (Vector Laboratories, Burlingame, CA, USA) in PBS for 30 min and then incubated with an antibody to beta-III tubulin (ab52623; Abcam) (1:500) overnight at 4°C. The cells were subsequently washed with PBS and incubated with an Alexa Fluor 488-conjugated secondary antibody (ab150081; Abcam) (1:500) for 1 hour in the dark. Signals were analyzed with ImageJ software (NIH, Bethesda, MD, USA) according to a previous report [20]. The average length of the projections of 45 DRG neurons from eight wells was calculated for each group. The same procedure was used to evaluate the effect of heated ADSC-CEs.

Length of Neurites of PC12D Cells

PC12D cells (provided by the Department of Cell Biology, Aichi Medical University School of Medicine) were seeded into 75 cm² flasks (1.0 × 10⁶ cells in 10 ml of medium) and cultured at 37°C under a 5% CO₂/95% air atmosphere. The basic medium consisted of DMEM/F12 supplemented with 10% FBS and 1% penicillin/streptomycin. The medium was changed twice a week, and the cells were passaged with 0.05% trypsin solution when they reached approximately 80% confluence.

PC12D cells were cultured in medium (DMEM/F12 supplemented with 10% FBS and 1% penicillin/streptomycin) supplemented with beta-NGF (R&D Systems, Minneapolis, MN, USA) (50 ng/ml). It was confirmed that the addition of beta-NGF caused these cells to elongate their processes. PC12D cells were seeded into 96-well plates (NIPPON Genetics) at 4.0 × 10^3^ cells/well in 100 µl of medium and cultured in basic medium. After 24 hours of culture, the medium was replaced with 100 µl of basic medium (control), basic medium containing beta-NGF (added to a concentration of 50 ng/ml), or basic medium containing ADSC-CEs (added to a protein concentration of 12.5 µg/ml). The cells were observed under the phase-contrast microscope on days zero, two, and five of culture, and the length of processes was measured using ImageJ software (NIH). Five wells were analyzed, and the average length of the longest PC12D cell processes in each well was calculated. The effect of heated ADSC-CEs was evaluated using the same procedure.

Statistical analysis

All quantitative data are expressed as the mean ± standard deviation. Comparisons between multiple groups were analyzed with unpaired analysis of variance (ANOVA) followed by multiple comparison test (Dunnett's test), and comparisons between two groups were analyzed using an unpaired t-test. Statistical software (ystat 2018; Shinya Yamazaki, Koriyama, Japan) was used for data analysis. A difference was considered significant when p < 0.05.

## Results

The multipotent differentiation potential of ADSCs

**Figure 2:**
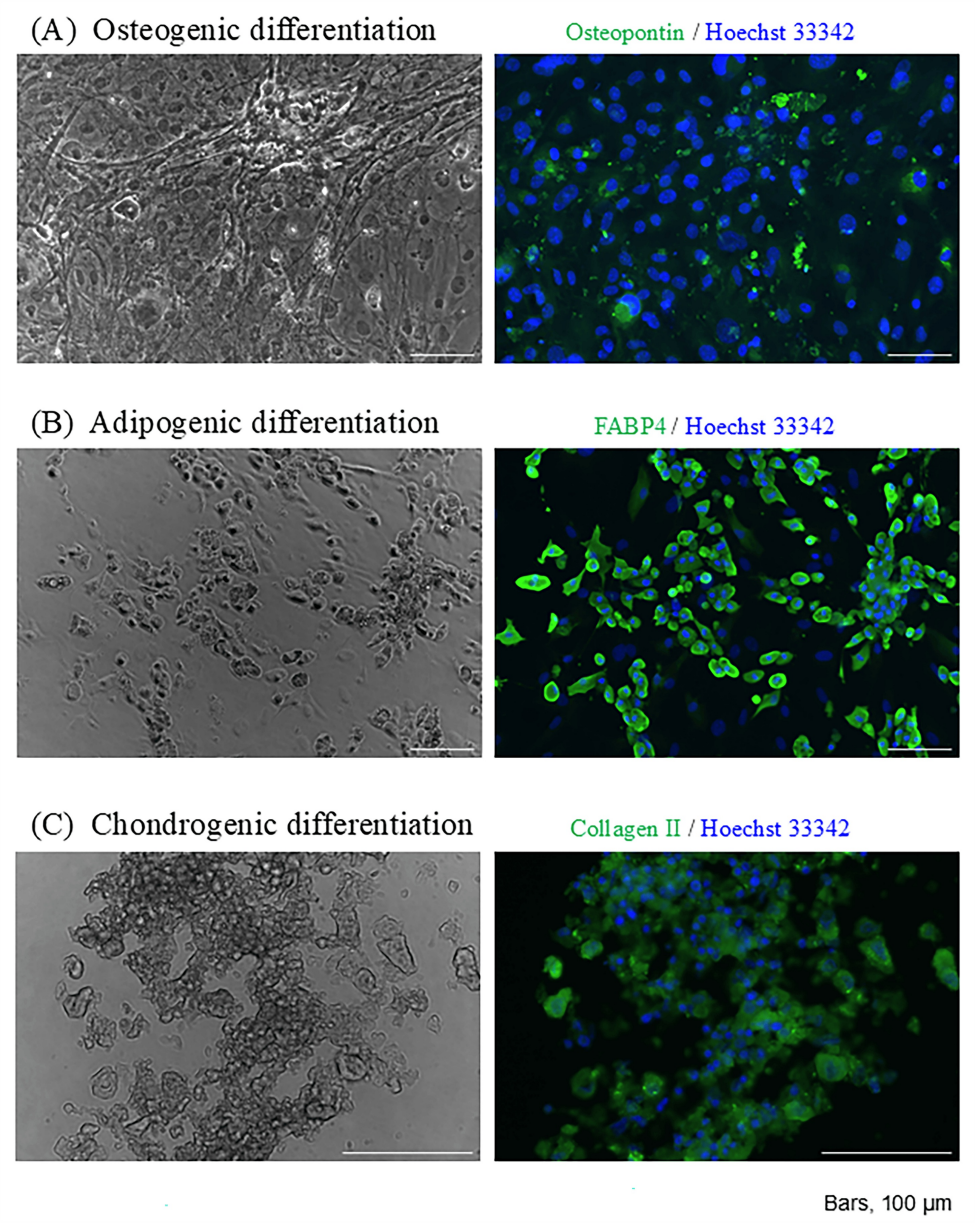
The multipotent differentiation potential of ADSCs The image is created by the author. The pluripotency of adipose-derived stem cells (ADSCs) was evaluated using the Mouse Mesenchymal Stem Cell Function Identification Kit (R&D Systems, Minneapolis, Minnesota, USA) in accordance with the manufacturer’s instructions. ADSCs showed osteopontin expression on day 21 of bone differentiation induction. Scale bar = 100 µm. ADSCs showed FABP4 expression on day 14 of adipocyte differentiation induction. Scale bar = 100 µm. ADSCs showed collagen II expression on day 21 of chondrocyte differentiation induction. Scale bar = 100 µm.

ADSCs showed osteopontin expression on day 21 of bone differentiation induction (Figure 2a). ADSCs showed FABP4 expression on day 14 of adipocyte differentiation induction (Figure 2b). ADSCs showed collagen II expression on day 21 of chondrocyte differentiation induction (Figure 2c).

Total protein concentration of ADSC-CEs

The total protein concentration in ADSC-CEs was 4,078 ± 74 µg/ml. The total protein concentration in heated ADSC-CEs was 600 ± 29 µg/ml. Heat treatment significantly reduced total protein concentration (p < 0.01, unpaired t-test).

ADSC-CEs increase the number of Schwann cells

**Figure 3:**
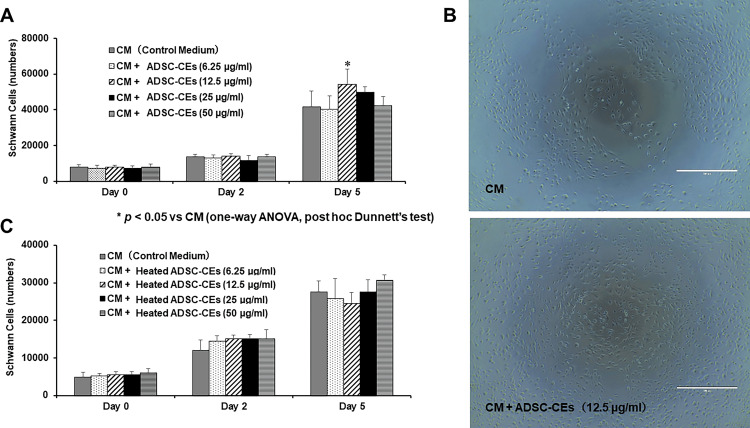
Effect of ADSC-CEs on Schwann cell proliferation The image is created by the author. ADSC-CEs: Adipose-derived stem cells cell extracts (A) The number of Schwann cells in control medium (CM) or medium containing ADSC-CEs was assessed by MTT assay. On day five, a significant increase in the number of Schwann cells was observed in the CM + ADSC-CEs (12.5 µg/ml) group (ADSC-CEs added to the medium to a protein concentration of 12.5 µg/ml) compared with the group cultured in CM. All data are expressed as mean ± standard deviation. *p < 0.05 vs. CM (one-way ANOVA, post hoc Dunnett’s test). (B) Schwann cells observed by phase-contrast microscopy. Top row: Schwann cells cultured in CM; bottom row: Schwann cells cultured in CM + ADSC-CEs (12.5 µg/ml). More Schwann cells were observed in the group cultured with CM + ADSC-CEs (12.5 µg/ml). Scale bar = 100 µm. (C) The number of Schwann cells cultured in CM or medium with heated ADSC-CEs was determined by MTT assay. There was no significant difference in the number of Schwann cells in the CM + heated ADSC-CEs group compared with the CM group. All data are expressed as mean ± standard deviation.

Figure 3 shows the effect of ADSC-CEs on the proliferation of Schwann cells. On days zero and two, there was no difference in the number of Schwann cells among the groups. On day five, a significant increase in the number of Schwann cells was observed in the CM + ADSC-CEs (12.5 µg/ml) group compared with the group cultured in control medium (Figure 3A). A greater number of Schwann cells was observed in the ADSC-CEs group in phase-contrast micrographs (Figure 3B). When heated ADSC-CEs were added to the medium, no difference in the number of Schwann cells was observed on days zero, two, or five (Figure 3C).

ADSC-CEs increase GFAP expression in Schwann cells

**Figure 4:**
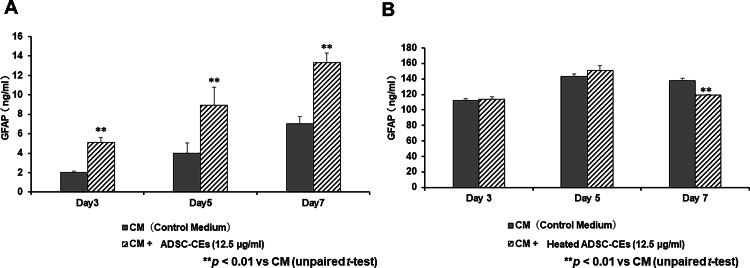
Effect of ADSC-CEs on glial fibrillary acidic protein (GFAP) expression in Schwann cells The image is created by the author. (A) GFAP expression in Schwann cells cultured in CM or medium containing ADSC-CEs was measured by ELISA. On days three, five, and seven, GFAP expression was significantly increased in the CM + ADSC-CEs (12.5 µg/ml) group compared with the group cultured in CM. All data are expressed as mean ± standard deviation. **p < 0.01 vs. CM (unpaired t-test). (B) GFAP expression in Schwann cells cultured in control medium (CM) or medium containing heated ADSC-CEs was measured by ELISA. On day seven, GFAP expression was significantly decreased in the CM + heated ADSC-CEs group compared with the CM group. All data are expressed as mean ± standard deviation. **p < 0.01 vs. CM (unpaired t-test).

Figure 4 shows the effect of ADSC-CEs on Schwann cell GFAP expression. On days three, five, and seven, Schwann cell GFAP expression was significantly increased in the CM + ADSC-CEs (12.5 µg/ml) group compared with the group cultured in control medium (Figure 4A). In contrast, GFAP expression in Schwann cells in the group cultured in medium containing heated ADSC-CEs was significantly decreased on day seven compared with the group cultured in control medium (Figure 4B).

ADSC-CEs promote neurite outgrowth in DRG neurons

**Figure 5:**
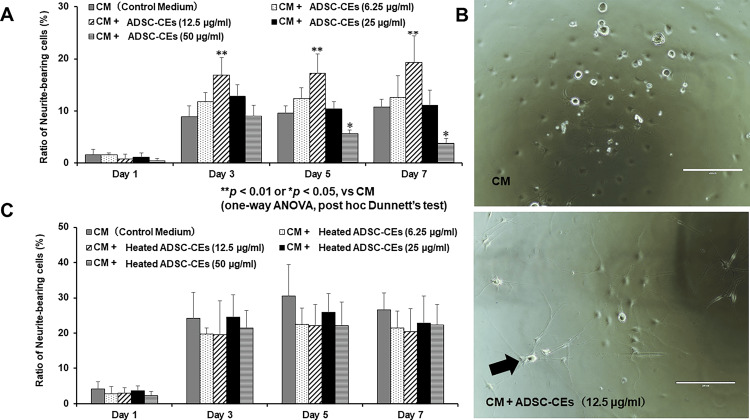
Effect of ADSC-CEs on the percentage of neurite-bearing cells The image is created by the author. (A) The percentage of neurite-bearing DRG neurons (those with neurites longer than the cell body) cultured in control medium (CM) or in medium containing ADSC-CEs. On days three, five, and seven, the percentage of neurite-bearing cells was significantly increased in the CM + ADSC-CEs (12.5 µg/ml) group compared with the group cultured in CM. On days five and seven, the percentage of neurite-bearing cells was significantly decreased in the CM + ADSC-CEs (50 µg/ml) group compared with the group cultured in CM. All data are expressed as mean ± standard deviation. **p < 0.01, *p < 0.05 vs CM (one-way ANOVA, post hoc Dunnett’s test). (B) DRG neurons observed by phase-contrast microscopy. Top row: DRG neurons cultured in CM. Bottom row: DRG neurons cultured in CM + ADSC-CEs (12.5 µg/ml). Arrows indicate neurite-bearing cells. More neurite-bearing cells were observed in the group cultured with CM + ADSC-CEs (12.5 µg/ml). Scale bar = 200 µm. (C) The percentage of neurite-bearing DRG neurons cultured in control medium (CM) or medium containing heated ADSC-CEs. There was no significant difference in the percentage of neurite-bearing cells in the CM + Heated ADSC-CEs group compared with the CM group. All data are expressed as mean ± standard deviation.

Next, we investigated the effect of ADSC-CEs on neuritic extension in DRG neurons. On days three, five, and seven, the percentage of neurite-bearing cells was significantly increased in the CM + ADSC-CEs (12.5 µg/ml) group compared with the group cultured in the control medium (Figure 5A). On days five and seven, the percentage of neurite-bearing cells was significantly decreased in the CM + ADSC-CEs (50 µg/ml) group compared with the group cultured in control medium (Figure 5A). In the phase-contrast micrographs, many neurite-bearing cells were observed in the group incubated with ADSC-CEs (Figure 5B). There was no difference in the percentage of neurite-bearing cells between the control group and the group in which heated ADSC-CEs were added (Figure 5C).

**Figure 6:**
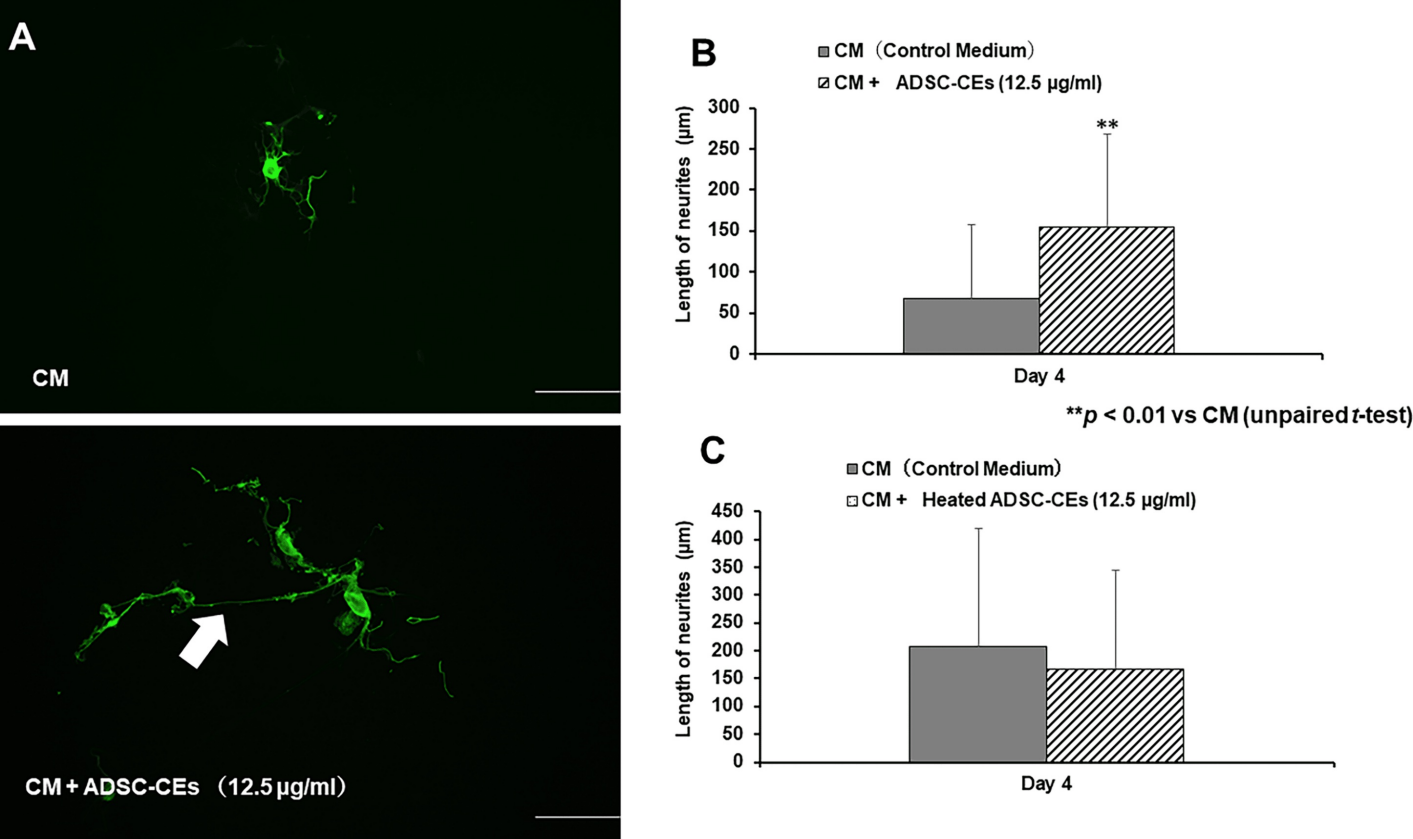
Effects of ADSC-CEs on the length of neurites in DRG neurons DRG neurons immunostained for beta-tubulin. Top row: DRG neurons cultured in control medium (CM). Bottom row: DRG neurons cultured in CM + ADSC-CEs (12.5 µg/ml). DRG neurons with long neurites (indicated by arrows) were observed in the CM + ADSC-CEs (12.5 µg/ml) group. Scale bar = 100 µm. The lengths of neurites of DRG neurons cultured in medium with CM or medium containing ADSC-CEs. On day 4, the length of the neurites of DRG neurons was significantly increased in the CM + ADSC-CEs (12.5 µg/ml) group compared with the CM group. All data are expressed as mean ± standard deviation. **p < 0.01 vs CM. The lengths of neurites of DRG neurons cultured in CM or medium containing heated ADSC-CEs. There was no significant difference in the length of elongated neurites of DRG neurons in the CM + Heated ADSC-CEs group compared with the CM group. All data are expressed as mean ± standard deviation.

Figure 6A shows DRG neurons immunostained for beta-tubulin. DRG neurons with long neurites were detected in the CM + ADSC-CEs (12.5 µg/ml) group. On day four, the neurite length in DRG neurons was significantly increased in the CM + ADSC-CEs (12.5 µg/ml) group compared with the group cultured in control medium (Figure 6B). No significant differences in neurite length were observed between the groups in which heated ADSC-CEs were added to the medium and the control group (Figure 6C).

ADSC-CEs promote neurite outgrowth in PC12D cells

**Figure 7:**
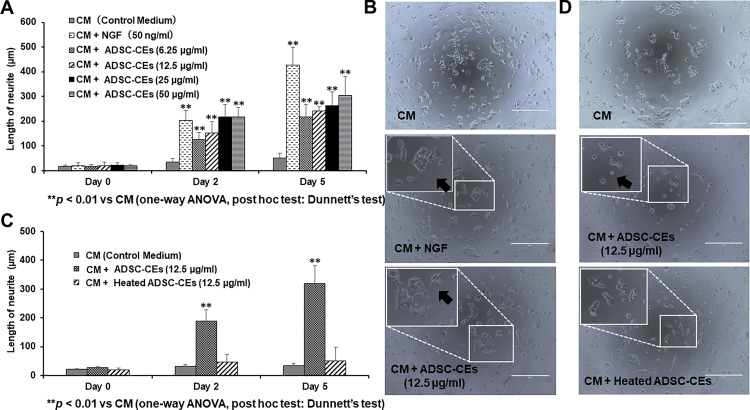
Effect of ADSC-CEs on the length of neurites in PC12D cells (A) The lengths of neurites of PC12D cells cultured in CM or medium containing ADSC-CEs. On days 2 and 5, neurite lengths were significantly increased in the CM + NGF (50 ng/ml) group (NGF added to the CM to a protein concentration of 50 ng/ml) compared with the group cultured in CM. On days two and five, neurite lengths were significantly increased in all groups cultured in medium containing ADSC-CEs compared with the group cultured in CM. All data are expressed as mean ± standard deviation. **p < 0.01 vs. CM (one-way ANOVA, post hoc Dunnett’s test). (B) PC12D cells observed by phase-contrast microscopy. Top row: PC12D cells cultured with CM. Middle row: PC12D cells cultured in CM + NGF. Bottom row: PC12D cells cultured in CM + ADSC-CEs. More neurites (indicated by arrows) were observed in the CM + ADSC-CEs group. Scale bar = 400 µm. (C) The lengths of neurites of PC12D cells cultured in CM or medium containing heated ADSC-CEs. On days two and five, neurite lengths were significantly increased in the CM + ADSC-CEs (12.5 µg/ml) group compared with the group cultured in CM. There was no significant difference in the length of neurites in the CM + heated ADSC-CEs group compared with the CM group. All data are expressed as mean ± standard deviation. **p < 0.01 vs. CM (one-way ANOVA, post hoc Dunnett’s test). (D) PC12D cells observed by phase contras microscopy. Top row: PC12D cells cultured in CM. Middle row: PC12D cells cultured in CM + ADSC-CEs (12.5 µg/ml). Bottom row: PC12D cells cultured in CM + heated ADSC-CEs. Few elongated neurites were observed in the CM + Heated ADSC-CEs group. Scale bar = 400 µm.

The effect of ADSC-CEs on neurite elongation was evaluated in PC12D cells. On day 0, there was no significant difference in the length of PC12D neurites between the groups. On days two and five, the length of neurites was significantly increased in all groups treated with ADSC-CEs compared with the group cultured in control medium (Figure 7A). In phase-contrast micrographs, many elongated neurites were detected in cells treated with ADSC-CEs (Figure 7B). No difference in neurite length was found between the control group and the group treated with heated ADSC-CEs (Figure 7C). Fewer neurites were observed in the group treated with heated ADSC-CEs (Figure 7D).

## Discussion

The strength of this study is the use of three different neural cell types, Schwann cells, DRG neurons, and PC12D cells, to assess the effects of ADSC-CEs. Immortalized Schwann cells have high proliferative capacity and are widely used in models of peripheral nerve regeneration to study the biocompatibility of novel materials and the activation of signaling pathways in vitro [22]. GFAP is an intermediate filament protein that is used as a marker of Schwann cell activation in nerve regeneration studies [23]. Sango et al. investigated the effects of ciliary neurotrophic factor (CNTF) on DRG neurons, and process extension was evaluated by assessing the percentage of neurite-bearing cells or by fluorescence immunostaining to assess the length of projections in vitro [20]. PC12 cells (a cell line derived from rat pheochromocytoma) and PC12D cells (a PC12 subline) exhibit cell growth arrest and differentiation into sympathetic neuron-like cells upon addition of NGF [24]. PC12 and PC12D cells are used to study the effects of compounds on the induction of neuronal differentiation [24]. In the present study, the addition of ADSC-CEs to the culture medium increased proliferation and GFAP expression in Schwann cells and increased the length of projections in DRG neurons and PC12D cells. The positive effects of ADSC-CEs in these three neural cell types, widely used in peripheral nerve regeneration studies, suggest that ADSC-CEs may be useful in peripheral nerve regeneration strategies involving Schwann cells and neurons.

Sowa et al. showed that transplantation of ADSCs into the lesion site promotes axonal regeneration in an animal model of sciatic nerve injury without these cells differentiating into Schwann cells [9]. Furthermore, they reported that ADSCs produce brain-derived neurotrophic factor (BDNF), nerve growth factor (NGF), and VEGF, which promote axon elongation and Schwann cell proliferation [10]. In a study investigating the benefits of ADSC-CEs in a corpus cavernosum nerve injury model, Maarten et al. found that the mechanism of recovery involved cell protection through neuron preservation and inhibition of apoptosis by ADSC-CEs [25]. These observations suggest that both ADSCs and ADSC-CEs promote nerve regeneration. Fang et al. reported that ADSC-CEs contain various growth factors and that intravenous administration of ADSC-CEs restores salivary gland secretory function in an animal model of salivary gland damage after irradiation [15]. They also reported that intravenous administration of bone marrow-derived CE, inactivated by heating, abolished its ability to promote functional recovery, suggesting that bone marrow-derived CE contains heat-labile active components that promote salivary gland regeneration [18]. In this study, similar to Fang et al. [18], heat treatment of ADSC-CEs reduced their total protein concentration. Notably, the heat treatment abolished the effects of the extract on Schwann cell proliferation, GFAP expression, and neurite growth in DRG neurons and PC12D cells. These findings suggest that heat-labile bioactive components in ADSC-CEs may promote peripheral nerve regeneration.

There are some limitations to this study. First, we did not investigate the bioactive components that promote peripheral nerve regeneration. Analysis of the proteins in ADSC-CEs revealed the presence of angiogenesis-related factors, including VEGF, HGF, and b-FGF (data not shown). However, it is unclear which growth factors (alone or in combination) are effective. Fang et al. found it necessary to use specific factor-depleted CE preparations to investigate the bioactive components using neutralizing antibodies against growth factors and cytokines [18]. Therefore, a similar method might be needed to investigate the bioactive components in ADSC-CEs in future studies. Second, we did not perform in vivo analyses. Previous studies on the effects of stem cells on peripheral nerve regeneration include a report on the neuroregenerative effects of human sweat gland-derived stem cells [26] and a report on the biocompatibility and neuroregenerative effects of ADSCs seeded on scaffolds prepared with ε-caprolactone and D,L-lactic acid, both in vitro only [27]. Therefore, in the present study, we investigated the effects of ADSC-CEs on peripheral neurons in vitro. In comparison, Fang et al. [15] evaluated the effects of CEs on salivary gland regeneration both in vitro and in vivo. Thus, future studies are needed to investigate the effects of ADSC-CEs on peripheral nerves in vivo, particularly to lay the foundation for translation to clinical applications. In addition, we used immortalized Schwann cells in this study because the proliferation assay performed on these cell lines is reliable, as these cells are not affected by cellular senescence, unlike primary cultured Schwann cells [22]. However, many immortalized cell lines are derived from tumor tissues, and their properties differ from those of normal tissues and cells [22]. Therefore, to better assess the effects of ADSC-CEs on neural cells, it will be necessary, in future studies, to perform in vitro and in vivo experiments using primary cell cultures.

## Conclusions

Peripheral nerve damage can cause pain and sensory impairment, severely reducing the quality of life. ADSC-CEs might be useful for regenerative therapy for various tissues and organs, particularly as they have the advantages of low tumorigenicity and immunogenicity. In this study, we demonstrated, for the first time, that ADSC-CEs stimulate the proliferation of Schwann cells and elevate GFAP expression in these cells, and that they promote the elongation of DRG neuron and PC12D cell projections. Moreover, the bioactive factors in ADSC-CEs that stimulate neuronal process extension and Schwann cell proliferation appear to be heat-labile protein(s). Our findings suggest that ADSC-CEs may have clinical application in peripheral nerve regeneration.

## Figures and Tables

**Figure 8:**
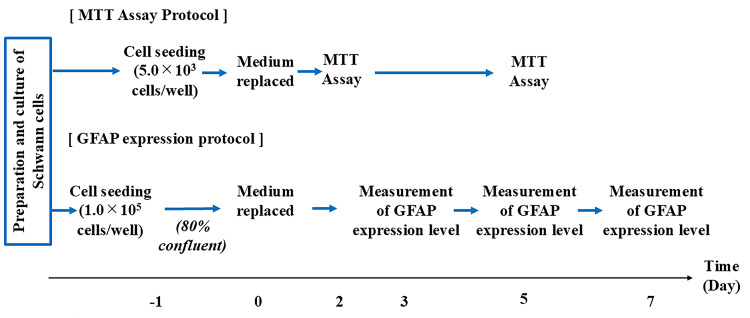
Experimental protocol overview diagram (Schwann cells)

**Figure 9:**
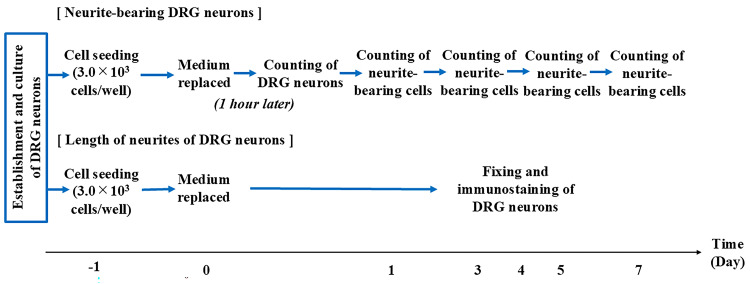
Experimental protocol overview diagram (DRG neurons)
